# Autocrine transforming growth factor β signaling regulates extracellular signal-regulated kinase 1/2 phosphorylation via modulation of protein phosphatase 2A expression in scleroderma fibroblasts

**DOI:** 10.1186/1755-1536-3-25

**Published:** 2010-12-06

**Authors:** Glady H Samuel, Andreea M Bujor, Sashidhar S Nakerakanti, Faye N Hant, Maria Trojanowska

**Affiliations:** 1 Arthritis Center, Division of Rheumatology, Boston University Medical Campus, Boston, MA, USA; 2 Division of Rheumatology and Immunology, Medical University of South Carolina, Charleston, South Carolina, USA

## Abstract

**Background:**

During scleroderma (SSc) pathogenesis, fibroblasts acquire an activated phenotype characterized by enhanced production of extracellular matrix (ECM) and constitutive activation of several major signaling pathways including extracellular signal-related kinase (ERK1/2). Several studies have addressed the role of ERK1/2 in SSc fibrosis however the mechanism of its prolonged activation in SSc fibroblasts is still unknown. Protein phosphatase 2A (PP2A) is a key serine threonine phosphatase responsible for dephosphorylation of a wide array of signaling molecules. Recently published microarray data from cultured SSc fibroblasts suggests that the catalytic subunit (C-subunit) of PP2A is downregulated in SSc. In this study we examined the role and regulation of PP2A in SSc fibroblasts in the context of ERK1/2 phosphorylation and matrix production.

**Results:**

We show for the first time that PP2A mRNA and protein expression are significantly reduced in SSc fibroblasts and correlate with an increase in ERK1/2 phosphorylation and collagen expression. Furthermore, transforming growth factor β (TGFβ), a major profibrotic cytokine implicated in SSc fibrosis, downregulates PP2A expression in healthy fibroblasts. PP2A-specific small interfering RNA (siRNA) was utilized to confirm the role of PP2A in ERK1/2 dephosphorylation in dermal fibroblasts. Accordingly, blockade of autocrine TGFβ signaling in SSc fibroblasts using soluble recombinant TGFβ receptor II (SRII) restored PP2A levels and decreased ERK1/2 phosphorylation and collagen expression. In addition, we observed that inhibition of ERK1/2 in SSc fibroblasts increased PP2A expression suggesting that ERK1/2 phosphorylation also contributes to maintaining low levels of PP2A, leading to an even further amplification of ERK1/2 phosphorylation.

**Conclusions:**

Taken together, these studies suggest that decreased PP2A levels in SSc is a result of constitutively activated autocrine TGFβ signaling and could contribute to enhanced phosphorylation of ERK1/2 and matrix production in SSc fibroblasts.

## Introduction

Scleroderma (SSc) is an autoimmune connective tissue disease characterized by excess production and deposition of extracellular matrix proteins leading to fibrosis of the tissue. During this process, normal fibroblasts become 'activated' and acquire a fibrotic phenotype. Transforming growth factor β (TGFβ) is a major profibrotic cytokine that plays important roles in a variety of physiological processes including cell proliferation, differentiation and survival. Although the mechanism of SSc fibrosis is not fully understood, there is strong evidence to suggest that TGFβ is central to the development and maintenance of the SSc phenotype [1-3]. Normal healthy dermal fibroblasts treated with TGFβ reproduce characteristics of SSc fibroblasts, further supporting the notion that TGFβ is a major mediator of SSc fibrosis [4].

During tissue injury, rapid release of TGFβ attracts inflammatory cells and fibroblasts to the site of injury, resulting in extracellular matrix production/remodeling and myofibroblast differentiation [5]. In normal tissue, following the injury response, coordinated apoptosis of fibroblasts and myofibroblasts prevents scarring and excessive fibrosis [6]. Published data suggests that normal and SSc dermal fibroblasts in culture secrete similar levels of TGFβ ligand [7,8] However, there is evidence of increased TGFβ signaling in SSc fibroblasts when compared to normal fibroblasts. Several studies have shown elevated levels of TGFβ receptors in SSc fibroblasts, which contribute to an autocrine TGFβ signaling cascade that is maintained in culture even in the absence of exogenous ligand [9-11]. The chronic activation of the TGFβ pathway in SSc produces fibroblasts with constitutively activated Akt and ERK1/2 pathways that are resistant to apoptosis [12-14]. The ERK1/2 pathway regulates numerous cellular processes and more recently has also been implicated in the process of fibrosis. Several papers have reported the function of the activated ERK1/2 pathway in fibrosis. For example, it has been demonstrated that the ERK1/2 pathway is required for Smad1 phosphorylation in response to overexpression of TGFβRI and for subsequent upregulation of connective tissue growth factor (CCN2) and other profibrotic genes [15]. Activation of mitogen-activated protein kinase kinase 1(MEK1)/ERK1/2 pathway was also shown to be a primary mechanism responsible for the TGFβ-induced upregulation of early growth response factor 1 (Egr-1) [16]. In addition, Chen *et al*. recently reported that activation of the ERK1/2 pathway contributes to the enhanced fibrosis and contractile ability of scleroderma fibroblasts [12]. The ERK1/2 pathway also induces up regulation of α_v_β_3 _integrin, which contributes to the autocrine TGFβ signaling in scleroderma fibroblasts [17]. However, although constitutively phosphorylated ERK1/2 may play important roles in SSc pathogenesis, the mechanism of prolonged activation of this pathway is largely unknown.

Protein phosphatase 2A (PP2A) is a member of the PPP family and one of the most abundant serine-threonine phosphatases, accounting for a substantial part of the total phosphatase activity. PP2A plays an important role in signal transduction pathways, regulation of cell cycle and transcriptional and translational regulation [18]. PP2A has a complex structure, comprising of three subunits: the catalytic (C), regulatory (B) and structural subunit (A). The catalytic subunit (C) and structural subunit (A) have two isoforms: α and β. The regulatory subunit (B) consists of four families with many isoforms that confer specificity of location and function [18]. The phosphatase activity of PP2A is present in the C-subunit and its effects include dephosphorylation of various transcription factors and protein kinases including MEK, ERK1/2, Akt, and sphingosine kinase (SK) [19-21]. Recently published microarray data from cultured early passage SSc fibroblasts suggests that the β isoform of the catalytic subunit of PP2A is downregulated in SSc [22]. Based on the evidence of constitutive activation of ERK1/2 pathways in SSc fibroblasts and recent microarray data suggesting that PP2A may also be altered in SSc, we wished to further study the mechanism and significance of dysregulated PP2A in SSc fibroblasts.

## Results

### TGFβ stimulates prolonged phosphorylation of ERK1/2 in dermal fibroblasts

**Figure 1:**
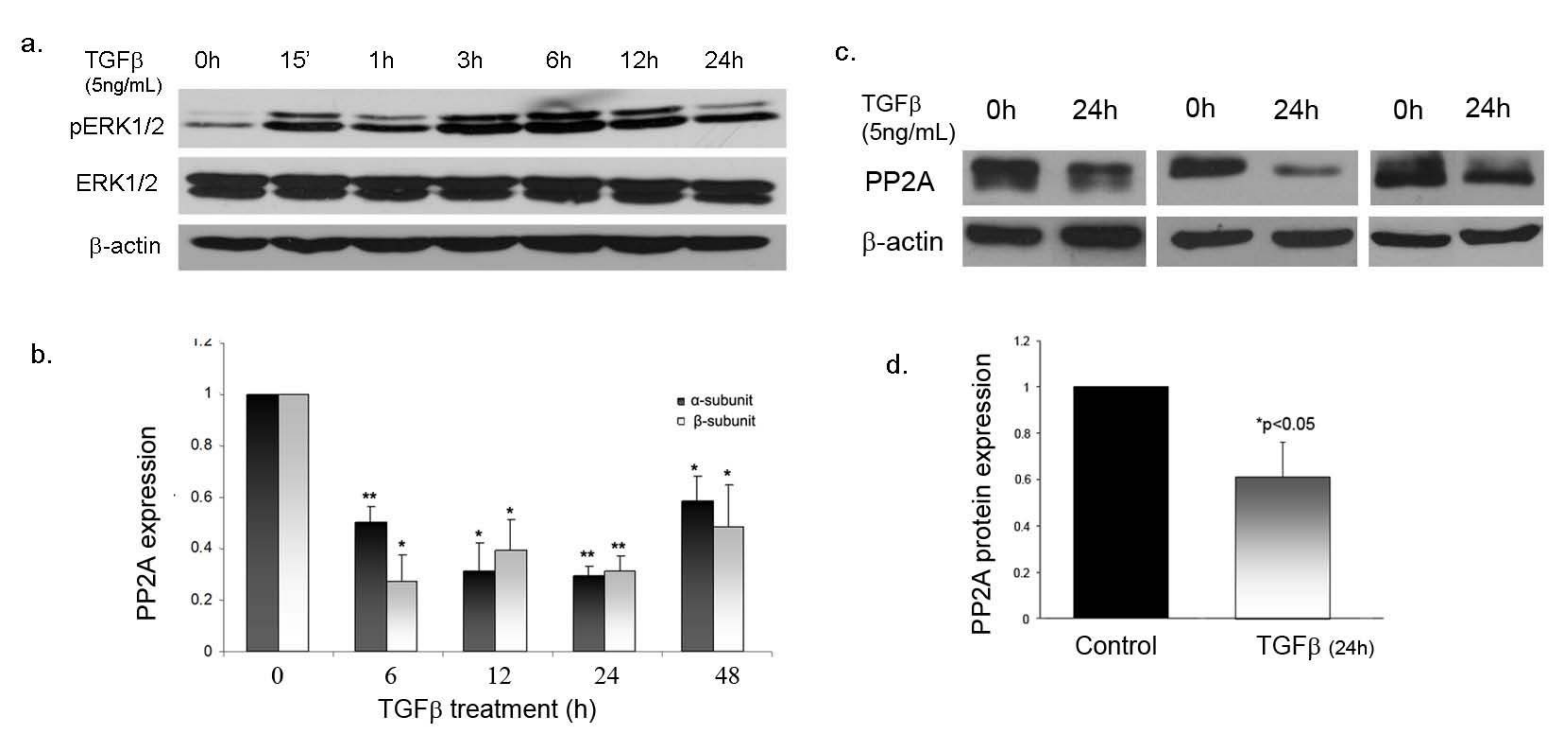
**Transforming growth factor (TGF)β regulates extracellular signal-regulated kinase (ERK)1/2 phosphorylation and protein phosphatase 2A (PP2A) expression in dermal fibroblasts**. **(a) **Adult dermal fibroblasts were treated with TGFβ (5 ng/ml) for 0 h, 15 min, 1 h, 3 h, 6 h, 12 h and 24 h after serum starvation. Western blot analysis was performed using phospho-ERK1/2 and ERK1/2 primary antibodies and rabbit horseradish peroxidase-conjugated secondary antibody. **(b) **Adult dermal fibroblasts were serum starved overnight and treated with 5 ng/ml of TGFβ for 0 h, 6 h, 12 h, 24 h and 48 h. RNA was then extracted and cDNA synthesized for quantitative PCR analysis; n = 3, *P < 0.05, **P < 0.01. **(c) **Dermal fibroblasts were treated for 24 h with 5 ng/ml of TGFβ after 24 h of serum starvation, and western blot analysis was performed using a PP2A antibody directed against the catalytic subunit; n = 5, representative blot of three experiments. **(d) **Bar graphs represent quantification of western blot analysis from (c); n = 5, *P < 0.05 versus normal control.

Because of the central role of TGFβ in the pathogenesis of SSc, we first examined the regulation of ERK1/2 phosphorylation by TGFβ treatment in healthy fibroblasts. To investigate the kinetics of ERK1/2 activation, time course experiments were performed. Near confluent cells were serum starved and then treated with TGFβ for increasing time periods ranging from 0-24 h. Using western blot analysis we observed that stimulation of cells with TGFβ resulted in rapid phosphorylation of ERK1/2 as early as 15 min and sustained ERK1/2 phosphorylation up to 24 h (Figure 1a). This suggests that TGFβ can activate both early and prolonged phosphorylation of ERK1/2 in dermal fibroblasts.

### PP2A expression is decreased upon treatment with TGFβ

Since PP2A has been previously described as a major ERK1/2 phosphatase we next sought to determine whether TGFβ could also be involved in the regulation of PP2A expression in dermal fibroblasts. Confluent dermal fibroblasts were serum starved and then treated with TGFβ for different time periods. The mRNA levels of α and β isoforms of the PP2A catalytic subunit were analyzed by quantitative reverse transcription (qRT)-PCR. As shown in Figure 1b, the mRNA levels of PP2A were decreased as early as 6 h after TGFβ addition and the lower levels persisted up to 48 h. Treatment of cells with TGFβ affected both catalytic subunit isoforms, but the β isoform showed a greater overall decrease. To further validate the effects of TGFβ on PP2A gene expression we measured the protein levels of PP2A (Figure 1c,d) after 24 h of TGFβ treatment. PP2A protein levels were decreased at 24 h, correlating with and confirming the mRNA data. These observations show that TGFβ negatively regulates PP2A expression, suggesting that PP2A may be involved in TGFβ-mediated ERK1/2 phosphorylation.

### PP2A inhibition contributes to increased ERK1/2 phosphorylation

**Figure 2:**
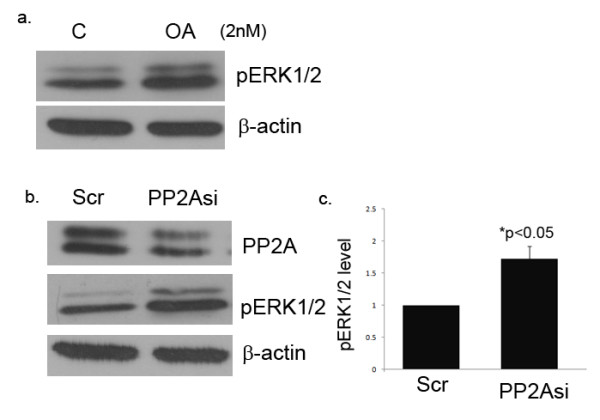
**Protein phosphatase 2A (PP2A) blockade stimulates extracellular signal-regulated kinase (ERK)1/2 phosphorylation in dermal fibroblasts**. **(a) **Dermal fibroblasts were treated with 2 nm of okadaic acid (OA) for 1 h and protein levels of phosphorylated ERK1/2 were measured by western blot. **(b) **Normal dermal fibroblasts were treated with 100 nm PP2A siRNA or scramble control for 48 h. Cell extracts were then analyzed for phosphorylated ERK1/2 after PP2A blockade. **(c) **Bar graph shows quantification of phospho-ERK1/2 after treatment with PP2A siRNA; *P *< 0.05. β Actin was used as the loading control.

To further confirm the role of PP2A in ERK1/2 phosphorylation in dermal fibroblasts, experiments were performed using okadaic acid (OA), a pharmacological inhibitor of PP2A activity, and PP2A-specific small interfering RNA (siRNA). Upon treatment of normal dermal fibroblasts with OA (2 nM) for 1 h, increased ERK1/2 phosphorylation was observed (Figure 2a). Consistent with this data, siRNA against the catalytic subunit of PP2A also increased phosphorylation levels of ERK1/2, suggesting that PP2A is involved in ERK1/2 dephosphorylation (Figure 2b,c). From these experiments we can conclude that PP2A downregulation in SSc fibroblasts may contribute to enhanced ERK1/2 phosphorylation. This data is in accordance with previously published reports that PP2A is an ERK1/2 phosphatase in several different cell types.

### PP2A expression is decreased and correlates with increased ERK1/2 phosphorylation in SSc dermal fibroblasts

**Figure 3:**
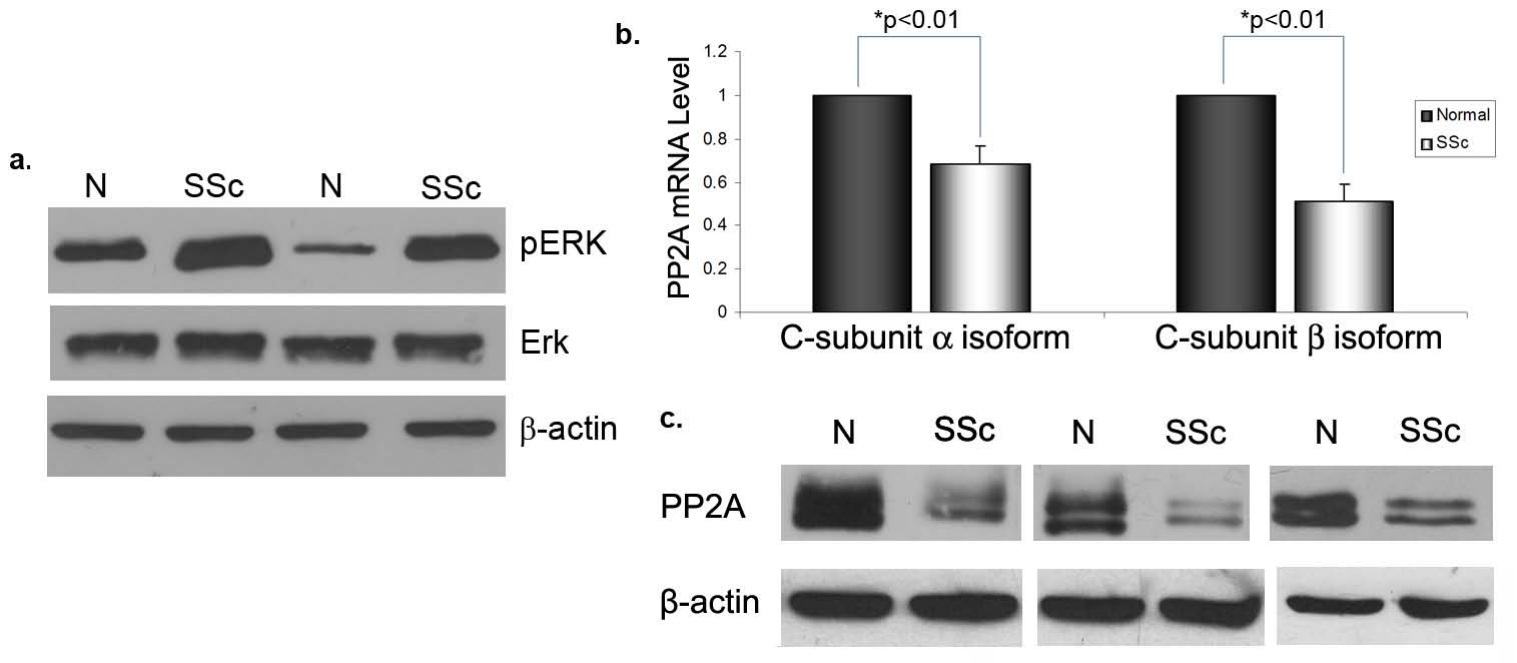
**Increased extracellular signal-regulated kinase (ERK)1/2 phosphorylation and decreased protein phosphatase 2A (PP2A) expression in scleroderma (SSc) fibroblasts**. Dermal fibroblasts obtained from SSc patients and matched controls were grown to confluence and then serum starved for 24 h. Cells were collected and in **(a) **the levels of ERK1/2 phosphorylation were analyzed by western blot. In **(b)**, the mRNA levels of the PP2A catalytic subunits α and β were analyzed using quantitative PCR. The mRNA values were normalized relative to matched controls (arbitrarily set as 1) and means ± standard error of the mean (SEM) of five independent experiments are shown (*P < 0.01). **(c) **The total protein levels of the PP2A catalytic subunit were measured by western blot. β Actin was used as a loading control.

To further study the relationship of PP2A and ERK1/2 phosphorylation in the pathological context, age, race and gender matched SSc and normal dermal fibroblasts obtained from patient biopsy were analyzed for PP2A expression and ERK1/2 activation. ERK1/2 phosphorylation was increased in SSc fibroblasts, consistent with data from previous reports [12] (Figure 3a). The mRNA levels of both isoforms of the PP2A C-subunit were significantly decreased in SSc fibroblasts when compared to normal controls (Figure 3b). Consistent with the mRNA data, the protein levels of the catalytic subunit of PP2A were significantly lower in SSc fibroblasts compared to normal controls (Figure 3c). The observations from SSc fibroblasts are consistent with the results from normal fibroblasts treated with TGFβ that show PP2A downregulation, suggesting a role for TGFβ in mediating these changes in SSc fibroblasts.

### Autocrine TGFβ signaling regulates PP2A expression in SSc fibroblasts

**Figure 4:**
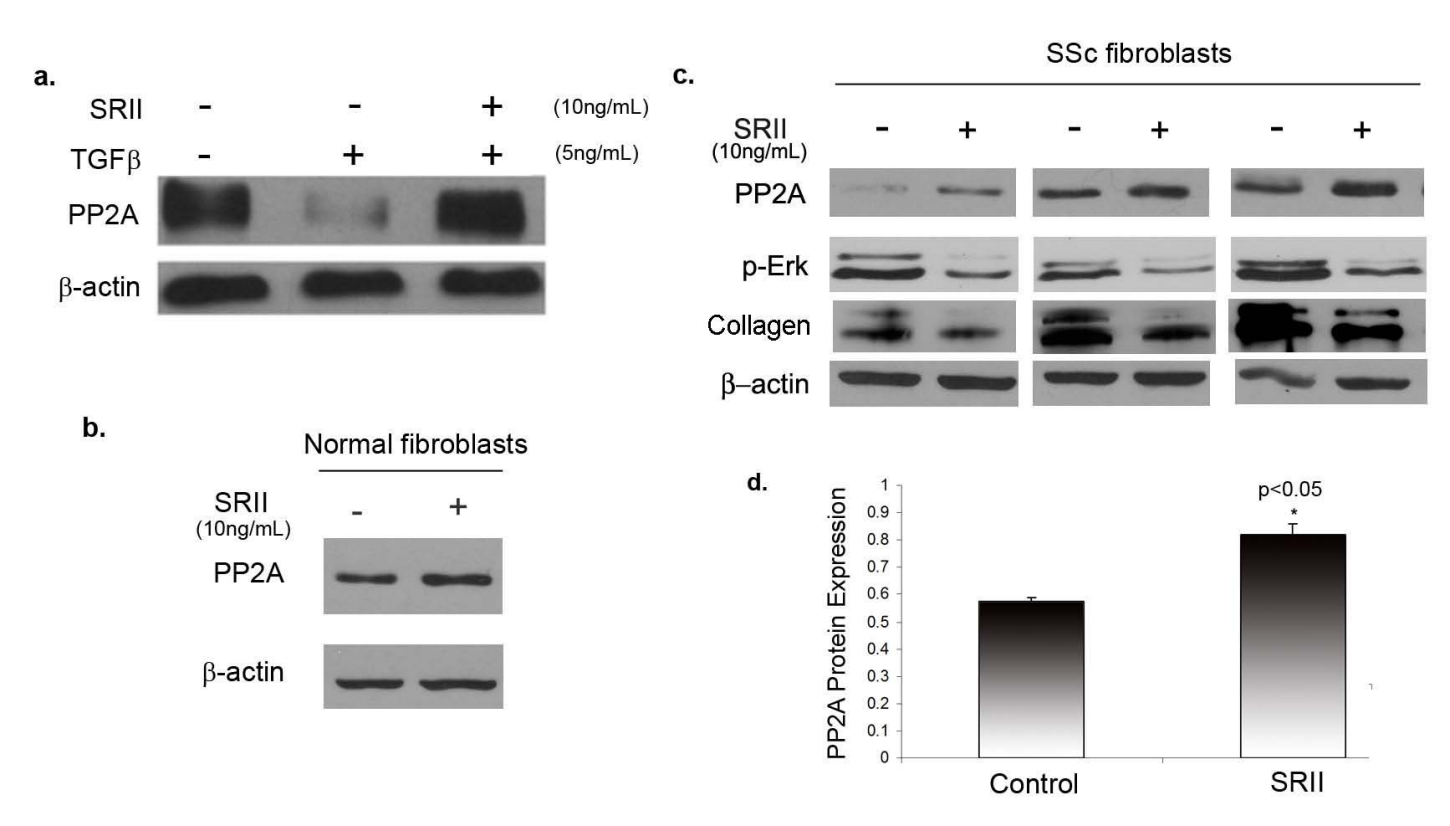
**Autocrine transforming growth factor (TGF)β signaling regulates protein phosphatase 2A (PP2A) expression in scleroderma (SSc) fibroblasts**. **(a) **Normal dermal fibroblasts were pretreated with recombinant transforming growth factor β soluble receptor II (SRII) (10 ng/ml) for 1 h and then treated with 5 ng/ml of TGFβ for 24 h. Western blot analysis was used to determine PP2A levels. **(b) **Normal and **(c) **SSc dermal fibroblasts were treated with SRII for 24 h after serum deprivation. Cells were collected and total protein levels of PP2A catalytic subunit were analyzed by western blot. In SSc cells, cell lysates were also analyzed for phospho-extracellular signal-regulated kinase (ERK)1/2 and type 1 collagen. β Actin was used as a loading control. **(d) **Bar graph shows quantification of PP2A protein expression after treatment of SSc fibroblasts with SRII; *P < 0.05.

Autocrine TGFβ signaling has been reported to play a major role in the pathogenesis of SSc. and blockade of endogenous TGFβ signaling has been shown to attenuate the scleroderma fibrotic phenotype [8]. Recombinant soluble TGFβ receptor II (SRII) has been successfully used as a TGFβ antagonist to block the effects of TGFβ signaling such as upregulated collagen production. SRII binds TGFβ ligand and prevents its interaction with surface receptors thereby neutralizing its activity [23,24]. To further investigate whether TGFβ signaling is responsible for the decreased levels of PP2A in SSc, we blocked autocrine TGFβ signaling using SRII. As a control experiment to confirm the effectiveness of SRII, normal cells were pretreated with SRII for 1 h and then treated with TGFβ for 24 h. Pretreatment with SRII efficiently blocked downregulation of PP2A by TGFβ (Figure 4a). Normal fibroblasts treated with SRII, showed no significant difference in basal PP2A expression (Figure 4b). We next determined the effects of SRII treatment on PP2A levels in SSc fibroblasts. Dermal fibroblasts obtained from three different SSc patient biopsies were grown to confluence, serum starved overnight and then treated with SRII for 24 h. The protein levels of PP2A were increased in SSc fibroblasts in the presence of SRII, suggesting that PP2A gene expression is regulated by the autocrine TGFβ signaling in these cells (Figure 4c). Increased expression of PP2A after SRII treatment was accompanied by a decrease in ERK1/2 phosphorylation and collagen expression (Figure 4c) providing evidence for a possible role for PP2A in the enhanced ERK1/2 phosphorylation and collagen expression observed in SSc.

### Blockade of ERK1/2 phosphorylation increases PP2A expression in SSc fibroblasts

**Figure 5:**
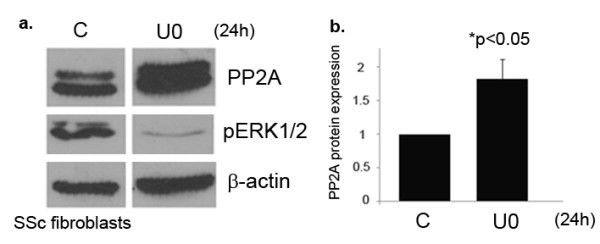
**Extracellular signal-regulated kinase (ERK)1/2 phosphorylation is a negative regulator of protein phosphatase 2A (PP2A) expression in scleroderma (SSc) fibroblasts**. **(a) **After treatment with ERK1/2 inhibitor U0126 (10 nm) for 24 h, PP2A expression was determined using western blot analysis in SSc fibroblasts; *P *< 0.05. **(b) **Bar graph showing quantification of PP2A expression from (a).

The activity of kinases and phosphatases is tightly regulated in the cell and often involve feedback mechanisms, which help maintain the levels of cellular phosphorylation. A study performed in human lung fibroblasts suggests that silencing of ERK1/2 is associated with a decrease in PP2A activity [25]. In order to further explore the relationship between PP2A and ERK1/2 phosphorylation, we examined the possibility that ERK1/2 activation could play a role in regulating the PP2A levels in SSc fibroblasts. SSc and normal dermal fibroblasts were treated with the pharmacological inhibitor U0126 to block ERK1/2 phosphorylation. Interestingly, only SSc fibroblasts showed increased PP2A expression upon treatment with U0126, suggesting that ERK1/2 activation contributes to maintaining decreased PP2A levels in SSc (Figure 5a,b). No significant change in PP2A levels in normal fibroblasts was observed (Data not shown). Taken together, these results suggest that autocrine TGFβ signaling in SSc fibroblasts leads to activation of ERK1/2 which in turn downregulates PP2A levels, thereby leading to even more prolonged phosphorylation of ERK1/2.

### PP2A is a negative regulator of collagen expression

**Figure 6:**
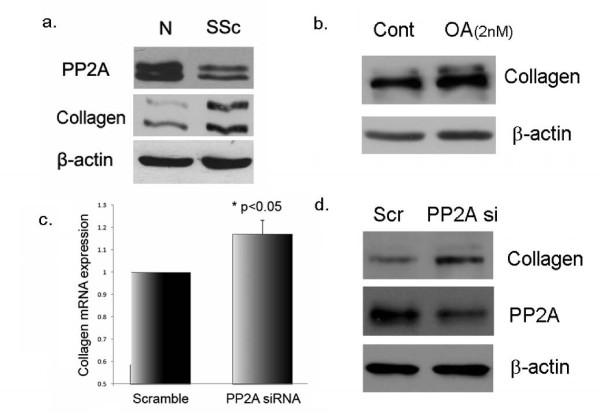
**Protein phosphatase 2A (PP2A) is a negative regulator of collagen expression**. **(a) **Extracts from normal and scleroderma (SSc) dermal fibroblasts were tested for PP2A and collagen expression using western blot analysis. β Actin was used as the loading control; n = 3, representative blot shown. **(b) **After treatment with okadaic acid (OA; 2 nM) collagen expression was determine using western blot analysis in SSc fibroblasts; n = 3, representative blot shown. **(c,d) **Normal dermal fibroblasts were treated with PP2A siRNA (100 nM) and collagen mRNA and protein expression measured. β Actin was used as the loading control; n = 3, representative blot shown.

Fibrosis is the hallmark of SSc fibroblasts and dysregulation of various signaling pathways have been implicated in increased collagen production in this disease. In SSc fibroblasts we observed an inverse correlation between PP2A levels and collagen expression (Figure 6a). To further determine whether PP2A blockade may contribute to increased collagen, normal dermal fibroblasts were treated with a specific pharmacological inhibitor of PP2A, OA (2 nm) for 24 h. This low dose of OA (2 nM) stimulated a modest increase in collagen protein levels (Figure 6b). However at higher doses we did not see this effect (data not shown). To further confirm the role of PP2A in regulation of collagen, we used siRNA specific for the catalytic subunit of PP2A. After PP2A blockade, in most cell lines tested we observed modest increases in mRNA and protein levels of collagen (Figure 6c,d).

## Discussion

In this study we have demonstrated that TGFβ is a negative regulator of PP2A. We found decreased expression of both the α and β isoform of the catalytic subunit of PP2A after TGFβ stimulation. To our knowledge this is the first report to demonstrate that TGFβ is a negative regulator of PP2A gene expression. In accordance with previously published studies our data also confirms that TGFβ treatment activates prolonged ERK1/2 phosphorylation. This study provides new evidence for the contribution of PP2A to the pathogenesis of SSc. Analysis of fibroblasts cultured from SSc skin biopsies shows decreased protein levels of the PP2A catalytic subunit, with downregulation of both α and β isoforms at the mRNA levels, reproducing the effects of TGFβ in normal dermal fibroblasts. These data validate a previous gene array study, which showed decreased levels of the β isoform in cultured SSc fibroblasts. However, downregulation of the α isoform, which is the most abundant isoform *in vivo*, has not been described in SSc fibroblasts. Previous reports indicated that an autocrine TGFβ signaling pathway contributes to the SSc phenotype [4]. We hypothesized that PP2A downregulation in SSc could be the result of constitutive TGFβ signaling. This hypothesis was supported by our data showing that recombinant soluble TGFβ receptor II, an antagonist of TGFβ signaling, was able to block the downregulation of PP2A and to reverse the constitutive phosphorylation of ERK1/2 in cultured SSc fibroblasts. This suggests that autocrine TGFβ signaling in SSc induces prolonged ERK1/2 phosphorylation, possibly via modulation of PP2A expression. Furthermore, in our study we observed that activated ERK1/2 can suppress PP2A expression in SSc fibroblasts but not in normal control fibroblasts. This suggested the presence of a self-sustained signaling loop between PP2A and ERK1/2 in SSc fibroblasts, whereby increased ERK1/2 phosphorylation in response to TGFβ downregulates PP2A expression and in turn results in a further increase in ERK1/2 phosphorylation. ERK1/2 phosphorylation has been previously implicated in fibrosis [12,26,27]. In this study, we observe that PP2A is also involved in regulation of collagen. The modest increase in collagen upon PP2A blockade suggests that the collagen production in SSc fibroblasts is a cumulative result of many dysregulated pathways present in SSc fibroblasts.

Reversible protein phosphorylation plays a central role in the regulation of vast majority of the biological processes. This process is tightly controlled by the protein kinases and phosphatases that together regulate the levels of cellular phosphoproteins. The balance between the activities of kinases and phosphatases is often disrupted during pathological conditions including neurodegenerative diseases and cancer [18,28]. Persistent downregulation of PP2A in SSc fibroblasts strongly suggests that this pathway is involved is the pathogenesis of SSc. It is noteworthy that the study of Tan and colleagues [22], who first reported on the aberrant expression of PP2A, was performed using fibroblasts from uninvolved skin. This suggests that this defect is present in the early stages of the disease. The constitutive activation of the ERK1/2 pathway in SSc may play a critical role in the development and maintenance of fibrosis and the activated status of explanted SSc fibroblasts. In addition to its role as a major ERK1/2 phosphatase, PP2A has been also implicated in the regulation of sphingosine kinase (SK), a profibrogenic sphingolipid enzyme induced by TGFβ [20,29]. SK catalyzes the conversion of sphingosine to sphingosine1 phosphate, which mimics some of the profibrotic effects of TGFβ [30,31]. Additionally, SK is a major prosurvival molecule and may also indirectly contribute to fibrosis by inducing resistance to apoptosis in activated fibroblasts [31]. Further experiments using animal models of PP2A knockout or transgenic mice would be essential to study and dissect the pathways involved in PP2A downregulation *in vivo *and its role in fibrosis. However there are several limitations to this approach considering the vast number of subunits and splice variants present for this molecule as well as the numerous substrates and methods of posttranslational regulation. Several experimental mouse models have been generated including the PP2AC knockout mouse and transgenic models of various other PP2A subunits [32]. The PP2Acα knockout mouse is embryonic lethal and results in degeneration of the embryo and lack of formation of the mesoderm. Interestingly, in these embryos, the two highly homologous catalytic subunits are found in different subcellular locations, the Cα in the plasma membrane and Cβ in the cytosol, making it unlikely that Cβ can compensate for Cα in these mice [32]. However, since these mice are embryonic lethal, a tissue-specific knockout of PP2Acα in fibroblasts would provide key insights into the role of PP2A in fibrosis.

## Conclusions

In conclusion, this study describes a novel role for TGFβ in the regulation of PP2A gene expression. While our study focused on ERK1/2, PP2A dephosphorylates numerous signaling molecules, many of them with a potential role in fibrosis, and it is likely that such global downregulation of PP2A activity would modulate additional cellular pathways. We also show that SSc fibroblasts have decreased levels of PP2A and that this could be restored by blockade of autocrine TGFβ signaling, suggesting that negative regulation of the PP2A catalytic subunit gene expression may be a physiological mechanism by which sustained ERK1/2 phosphorylation occurs in SSc. This study highlights an unanticipated regulatory function for TGFβ in modulating PP2A activity and provides support for an essential role of PP2A in the pathogenesis of SSc. Further studies are necessary to gain insight into the role of PP2A and ERK1/2 activation in the modulation of ECM components in SSc fibroblasts.

## Methods

### Reagents

The following antibodies were used: anti-PP2A (Upstate, Temecula, CA, USA), anti-phospho-ERK1/2, anti-ERK1/2, anti-Akt (Cell Signaling, Beverly, MA, USA), anti-phospho-Akt, Ser 473 (Santa Cruz Biotechnology, Santa Cruz, CA, USA), monoclonal β actin (Sigma Aldrich, St Louis, MO, USA), anti-type 1 collagen (Southern Biotech, Birmingham, AL, USA).

Recombinant human TGFβ1 was obtained from R&D Systems (Minneapolis, MN, USA). OA was purchased from Sigma Aldrich. Tissue culture reagents, Dulbecco's modified Eagle medium (DMEM) and 100× antibiotic antimycotic solution (penicillin streptomycin and amphotericin B) were obtained from Gibco BRL (Grand Island, NY, USA) and fetal bovine serum was purchased from HyClone (Logan, UT, USA). Enhanced chemiluminescence reagent and bovine serum albumin (BSA) protein assay reagent were obtained from Pierce (Rockford, IL, USA). TriReagent was purchased from the Molecular Research Center (Cincinnati, OH, USA). Primers were purchased from Operon (Huntsville, AL, USA). SMARTpool siRNA against PP2A C-subunit was purchased from Dharmacon RNA Technologies (Lafayette, CO, USA) and Hiperfect siRNA transfection reagent from Qiagen (Germantown, MD, USA).

### Cell culture

Human dermal fibroblast cultures were established from biopsy specimens obtained from the dorsal forearms of SSc patients with diffuse cutaneous disease and from age, race and gender matched healthy donors, upon informed consent and in compliance with the Institutional Review Board. Dermal fibroblasts were cultured from the biopsy specimens as described previously [15]. Normal and SSc skin fibroblasts were cultured in DMEM supplemented with 10% FBS and 1% antibiotic antimycotic solution. For experiments cells were pretreated with serum-free media for 24 h. Cells were treated with TGFβ, 5 ng/ml.

### Real-time PCR

Total RNA was isolated from dermal fibroblasts using TriReagent (Molecular Research Center) according to the manufacturer's instructions. RNA (2 μg) was reverse transcribed in a 20-μl reaction using random primers and Transcriptor First Strand synthesis kit (Roche Applied Sciences Indianapolis, IN. Quantitative (q)PCR was carried out using IQ SYBR Green mixture (Bio-Rad, Hercules, CA) on an iCycler PCR machine (Bio-Rad) using 1 μl of cDNA in triplicate with β actin as the internal control. The primers used are as follows. PP2A C-subunit α isoform: forward, 5'-GCACTTGATCGCCTACAAGA-3' and reverse, 5'-GAAATATCTTGCCCAAAGGTGT-3'. PP2A C-subunit β isoform: forward, 5'-TTCTTGTAGCATTAAAGGTGCGT-3' and reverse, 5'-CATTCCCATACTTCGCAGACA-3'.

### Immunoblotting

Whole cell protein extracts were prepared according to the manufacturer's recommendations (Pierce). Immunoblotting was performed as previously described [33].

### RNA interference

SMARTpool siRNA directed against human PP2A catalytic subunit was purchased from Dharmacon RNA Technologies. Negative-control siRNA was purchased from Qiagen (Chatsworth, CA, USA) and Hiperfect transfection reagent (Qiagen) was used for transfection of dermal fibroblasts according to the manufacturer's recommendations.

## Authors' contributions

GHS was involved in the development/experimental design of the project, performed the majority of the experiments, performed data acquisition and analysis, and wrote the manuscript. AMB isolated SSc and normal fibroblasts from patient biopsies used for experiments, and was involved with experimental design. SSN performed some inhibitor experiments and was involved with experimental design. FH provided biopsies from patients and was involved with experimental design. MT was involved with the conception, experimental design and supervision of the study. All authors have read and approved the final manuscript.
